# Causal analysis of body composition measurements in osteoarthritis knee: a two-sample mendelian randomization study

**DOI:** 10.1186/s12891-024-07465-3

**Published:** 2024-04-29

**Authors:** Guoxin Huang, Weimin Hong, Ke Wang, Ming Xu, BingQian Chen, Da Qian, Bin Pei

**Affiliations:** 1 Department of Evidence-Based Medicine Center, Xiangyang No.1 People’s Hospital, Hubei University of Medicine, Xiangyang, 441000 China; 2 School of Public Health and Management, Hubei University of Medicine, Shiyan, Hubei China; 3 General Surgery, Cancer Center, Department of Breast Surgery, Zhejiang Provincial People’s Hospital (Affiliated People’s Hospital), Hangzhou Medical College, Hangzhou, Zhejiang 310000 China; 4 Department of Burn and Plastic Surgery-Hand Surgery, Changshu Hospital Affiliated to Soochow University, Changshu No.1 People’s Hospital, Changshu, 215500 China; 5 Department of Orthopedics, Changshu Hospital Affiliated to Soochow University, Changshu No.1 People’s Hospital, Changshu, 215500 China

**Keywords:** Mendelian randomization, Meta-analysis, Osteoarthritis knee, Body composition measurements

## Abstract

**Background:**

To analyse the causal associations of different physical measures with osteoarthritis knee (KOA).

**Methods:**

Exposure factors (weight, body mass index (BMI), body fat percentage, waist circumference, hip circumference, waist–hip ratio (WHR), and basal metabolic rate (BMR)), and outcome factor KOA were analyzed by inverse-variance weighted (IVW) method, along with heterogeneity test, sensitivity and pleiotropy analyses. Meta-analysis was used to combine the effect values of IVW methods in different data sources.

**Results:**

Weight, BMI, body fat percentage, waist circumference, hip circumference and BMR analyses showed causal association with increased KOA risk, while WHR analysis indicated a reduction of the incidence of KOA. *P*-value for all the results was less than 0.05 and *F*-value large than 20. All results were negative for heterogeneity tests and sensitivity analyses, and there was pleiotropy in weight and BMR. Meta-analysis results showed that the results of Odds Ratios (95% Confidence Intervals) for Weight (1.43(1.35–1.51)), BMI (1.40(1.10–1.78)), body fat percentage (1.56(1.44–1.68)), waist circumference (1.40(1.10–1.78)), hip circumference (1.37(1.30–1.44)), WHR (0.86(0.71–1.04)) and BMR (1.36(1.27–1.46) were consistent with the ones by Mendelian randomization analyses.

**Conclusions:**

Body fat percentage may be a better indicator of KOA than BMI. In addition, weight and BMR may have a causal effect in KOA, but WHR does not have a causal relationship. BMI, body fat percentage, waist circumference, and hip circumference has a causal effect on KOA.

## Background

Osteoarthritis knee (KOA) is a chronic arthritic disease characterized by degenerative lesions and osteophytes in the knee cartilage [1, 2]. Clinical manifestations include pain, restricted motion, joint deformity, and bone friction sounds [3, 4]. With the population aging, the incidence of KOA is anticipated to rise from 13.8 to 15.7% by 2032, placing a huge burden on families and society [5, 6].

Several risk factors have been established to be caused with KOA, including age, gender, previous knee injury, occupational performance, and overweight or obesity. Overweight or obesity has been found to have a temporal causal association with the development and progression of KOA in early cohort studies [7–9]. Overweight or obesity is measured in various ways, such as body mass index (BMI), body fat percentage, waist circumference, hip circumference, waist–hip ratio (WHR), and basal metabolic rate (BMR). Although previous studies have found that all these indicators are risk factors for KOA, their causal relationship is not yet clear [10–12].

Mendelian randomization (MR) is a causal inference approach that uses genetic variation as an instrumental variable (IV); it is based on the principle of using the random division and combination of gametes during sexual reproduction to simulate the random assignment process to the subject of the study [13, 14]. Katan was the first to formulate a MR method for exploring the direct increase in cancer risk cause with low serum cholesterol levels [15]. In recent years, it has been widely used in the study of causal associations in a variety of diseases [16–18]. MR uses IVs, usually single nucleotide polymorphisms (SNPs), which are reliably caused with exposure and do not vary with caused lifestyle or socio-economic factors, and have the potential to confound traditional observational associations [19, 20]. Therefore, our study used MR to explore the causal relationship between weight, BMI, body fat percentage, waist circumference, hip circumference, WHR, and BMR in KOA. Data from multiple datasets for the same indicator were combined using meta-analysis. Through exploring the causal association between body composition measurements and KOA, it can help to make relevant interventions in the clinic to effectively prevent the development of KOA, and to make the patients with KOA have better regression.

## Methods

### Study design

**Fig. 1:**
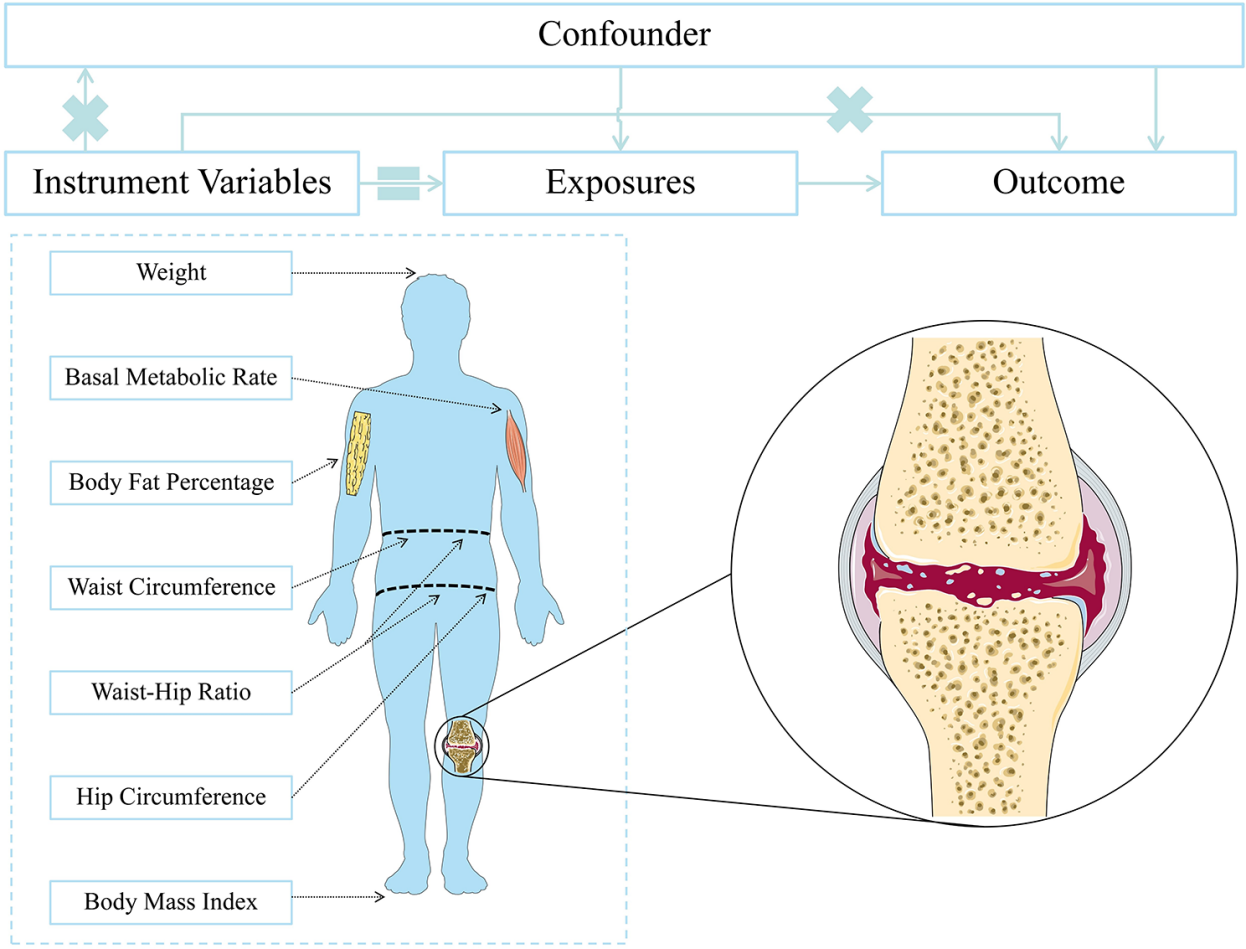
Overview of the design of this Mendelian randomization (MR) study on body composition measurements and osteoarthritis knee

This study used MR to explore the causal relationship between weight, BMI, body fat percentage, body fat percentage, waist circumference, hip circumference, WHR, and BMR in KOA. Three assumptions need to be met in order to minimise bias due to unobserved confounding, measurement error, and reverse causality. They are (1) relevance, where the IV is strongly correlated with the exposure factor; (2) independence, whereby the IV is not correlated with the confounding factor; and (3) exclusion restriction, there is no causal association between the instrument variable and outcome independent of the exposure [21]. An overview of the study design is shown in Fig. 1. This study is reported following the STROBE-MR guidelines.

### Data sources

All analysed data are available in the IEU OpenGWAS project for this study (https://gwas.mrcieu.ac.uk/). Exposure factors were body composition measurements, including weight, BMI, body fat percentage, waist circumference, hip circumference, WHR, and BMR. The principle of selection was that the same exposure factor was selected, systematic and comprehensive search for datasets on body composition measurements, with a screening process that: (1) has a clear data source (e.g., GIANT, MRC-IEU, Neale Lab, Within family GWAS, etc.); and (2) uses the most recent year of data for the same data source. SNPs were from individuals of European origin, including both males and females. The KOA outcome factor was derived from 29 999 696 SNPs obtained from 403 124 European populations, which were sequenced by Tachmazidou et al. and published in the UK Biobank consortium [22]. Detailed information is shown in Table 1.

**Table 1:** Overview of the data sources of the instrumental variables used in the MR study

Expose/outcome	Dataset	Year	Population	Sex	Sample size	Number of SNPs	Author	Consortium
Weight	ukb-b-11,842	2018	European	Both	461,632	9,851,867	Ben Elsworth	MRC-IEU
	ukb-a-249	2017	European	Both	336,227	10,894,596	Neale	Neale Lab
Body mass index	ieu-b-40	2018	European	Both	681,275	2,336,260	Yengo, L	GIANT
	ukb-b-19,953	2018	European	Both	461,460	9,851,867	Ben Elsworth	MRC-IEU
	ukb-a-248	2017	European	Both	336,107	10,894,596	Neale	Neale Lab
	ieu-b-4816	2022	European	Both	99,998	7,191,606	Howe LJ	Within family GWAS
Body fat percentage	ukb-b-8909	2018	European	Both	454,633	9,851,867	Ben Elsworth	MRC-IEU
	ukb-a-264	2017	European	Both	331,117	10,894,596	Neale	Neale Lab
Waist circumference	ukb-b-9405	2018	European	Both	462,166	9,851,867	Ben Elsworth	MRC-IEU
	ukb-a-382	2017	European	Both	336,639	10,894,596	Neale	Neale Lab
	ieu-a-61	2015	European	Both	232,101	2,565,408	Shungin D	GIANT
Hip circumference	ukb-b-15,590	2018	European	Both	462,117	9,851,867	Ben Elsworth	MRC-IEU
	ukb-a-388	2017	European	Both	336,601	10,894,596	Neale	Neale Lab
	ieu-a-49	2015	European	Both	213,038	2,559,739	Shungin D	GIANT
Waist-hip ratio	ieu-a-73	2015	European	Both	212,244	2,560,782	Shungin D	GIANT
	ieu-b-4830	2022	European	Both	85,978	7,908,954	Howe LJ	Within family GWAS
Basal metabolic rate	ukb-b-16,446	2018	European	Both	454,874	9,851,867	Ben Elsworth	MRC-IEU
	ukb-a-268	2017	European	Both	331,307	10,894,596	Neale	Neale Lab
Knee osteoarthritis	ebi-a-GCST007090	2019	European	Both	403,124	29,999,696	Tachmazidou I	UK Biobank

SNPs: single nucleotide polymorphisms, Both: Males and Female

### Genetic instrument selection

To avoid strong linkage disequilibrium between SNPs, then genome-wide significant SNPs with independent and highly correlated exposure factors, as well as outcome variables were selected as IVs. The genome-wide information from the Thousand Genomes Project was used as a reference to screen for IVs without linkage effects [23]: (1) the parameters of weight, BMI, body fat percentage, waist circumference, hip circumference, WHR, and BMR datasets with genome-wide significance were set to *P* < 5 × 10^− 8^; (2) the linkage disequilibrium parameter (r^2^) was set to 0.001; and (3) the genetic distance was set to 10 MB, to screen for IVs without linkage effects. Then, IVs that were apparently caused with KOA were excluded from the screened IVs (*P* < 0.05). At the same time, the data were pre-processed so as to ensure consistency in effects equivalence and effect sizes. Finally, the strength of causal association of the genetic instruments for each putative risk factor was quantified by the F statistic (F = β^2^/se^2^) for all SNPs, to assess the power of the SNPs, If the F-statistic is much greater than 10, the likelihood of weak IV bias is small [24].

### Statistical analysis

The MR analysis used the inverse-variance weighted (IVW), MR-Egger, weighted median, simple mode, and weighted mode methods. IVW method is the primary statistical method and the other four methods are supplementary statistical methods. Heterogeneity was tested by the IVW and MR-Egger methods. The leave-one-out method was applied to sensitivity analyses, which explored the effect of a single SNP on causal associations by excluding each SNP. MR pleiotropy test function was performed to ensure that the results were free of horizontal pleiotropy, the intercept term of the MR-Egger regression was used to test for the presence of pleiotropy, and when p-value > 0.05, it can be assumed that no pleiotropy exists. MR analysis was based on R (4.1.2) software, applying the “TwoSampleMR” package.

To increase the generalisability and persuasiveness of the results of the MR analyses, Meta- analyses were used to combine IVW values from different data sources. Meta-analysis was conducted by Stata (12.00) software to combine the odds ratio (OR) values of the IVW results. Heterogeneity between studies adopted *χ*^*2*^ test are combined with *I*^*2*^ for qualification. When *I*^*2*^ < 50% and *P* > 0.1, the heterogeneity between studies was small and a fixed-effects model was used for statistical analysis; otherwise, a random-effects model was used.

## Results

### Weight

The MRC-IEU consortium’s dataset was screened to select a total of 298 SNPs as IVs. The IVW results showed that weight was causally related to an increased risk of KOA (OR = 1.47, 95% confidence interval [CI] = 1.36–1.59). A total of 199 SNPs were included as IVs in the Neale Lab consortium’s dataset. The IVW results were consistent with the former (OR = 1.39, 95% CI = 1.28–1.51) (Table 2).

**Table 2:** Mendelian randomization results in causal association between weight and KOA

Expose	Consortium	SNPs	Method	OR (95%CI)	*P*_Effect_	*P*_Heterogeneity_	*P*_Intercept_	F _Statistic_
Weight	MRC-IEU	298	IVW	1.47(1.36–1.59)	1.38 × 10^− 21^	0.93	0.16	59.43
			MR Egger	1.25(0.98–1.58)	7.18 × 10^− 2^	0.93		
			Weighted median	1.56(1.38–1.75)	2.72 × 10^− 13^			
			Simple mode	1.98(1.35–2.89)	4.99 × 10^− 4^			
			Weighted mode	1.70(1.21–2.40)	2.45 × 10^− 3^			
	Neale lab	199	IVW	1.39(1.28–1.51)	1.16 × 10^− 14^	0.98	0.04	53.59
			MR Egger	1.08(0.84–1.38)	5.56 × 10^− 1^	0.99		
			Weighted median	1.43(1.26–1.62)	1.51 × 10^− 8^			
			Simple mode	1.79(1.25–2.56)	1.84 × 10^− 3^			
			Weighted mode	1.62(1.18–1.83)	3.43 × 10^− 3^			

SNPs: single nucleotide polymorphisms, OR: odds ratio (OR = 1 Exposure does not affect odds of outcome, OR > 1 Exposure caused with higher odds of outcome, OR < 1 Exposure caused with lower odds of outcome), CI: confidence interval, IVW: inverse-variance weighted. P _Effect_: Mendelian randomisation results in p-values; P _Heterogeneity_: Heterogeneity test; P _Intercept_: pleiotropy analysis; F _Statistic_: F values

### Body mass index

The dataset of MRC-IEU consortium’s dataset was filtered and included a total of 249 SNPs as IVs. The IVW results exhibited that BMI were determined to have a potential positive causal effect on KOA (OR = 1.55, 95% CI = 1.43–1.67). A total of 176 SNPs in the Neale Lab consortium’s dataset, 308 SNPs in the GIANT consortium’s dataset and 24 SNPs in the within family GWAS consortium’s dataset served as IVs, among which the IVW analyses showed similar results (OR = 1.56, 95% CI = 1.44–1.69), OR = 1.50, 95% CI = 1.39–1.62), and OR = 1.06, 95% CI = 1.04–1.10), respectively (Table 3).

**Table 3:** Mendelian randomization results in causal association between BMI and KOA

Expose	Consortium	SNPs	Method	OR (95%CI)	*P*_Effect_	*P*_Heterogeneity_	*P*_Intercept_	F _Statistic_
BMI	GIANT	308	IVW	1.50(1.39–1.62)	5.52 × 10^− 26^	0.98	0.81	66.29
			MR Egger	1.47(1.18–1.83)	7.51 × 10^− 4^	0.98		
			Weighted median	1.53(1.36–1.73)	2.76 × 10^− 12^			
			Simple mode	2.22(1.59–3.11)	4.17 × 10^− 6^			
			Weighted mode	1.62(1.28–2.04)	6.04 × 10^− 5^			
	MRC-IEU	249	IVW	1.55(1.43–1.67)	1.59 × 10^− 28^	1.00	0.23	57.03
			MR Egger	1.34(1.05–1.71)	2.10 × 10^− 2^	1.00		
			Weighted median	1.55(1.38–1.74)	2.28 × 10^− 13^			
			Simple mode	1.79(1.32–2.43)	2.06 × 10^− 4^			
			Weighted mode	1.60(1.24–2.07)	4.01 × 10^− 4^			
	Neale lab	176	IVW	1.56(1.44–1.69)	3.09 × 10^− 27^	1.00	0.21	49.95
			MR Egger	1.31(0.98–1.74)	6.56 × 10^− 2^	1.00		
			Weighted median	1.60(1.41–1.81)	4.85 × 10^− 14^			
			Simple mode	1.88(1.38–2.55)	8.30 × 10^− 5^			
			Weighted mode	1.65(1.27–2.15)	2.95 × 10^− 4^			
	Within family GWAS	24	IVW	1.07(1.04–1.10)	7.11 × 10^− 6^	0.89	0.95	49.77
			MR Egger	1.07(0.97–1.18)	1.91 × 10^− 1^	0.85		
			Weighted median	1.08(1.04–1.12)	1.44 × 10^− 4^			
			Simple mode	1.08(1.02–1.15)	2.33 × 10^− 2^			
			Weighted mode	1.08(1.02–1.14)	1.82 × 10^− 2^			

SNPs: single nucleotide polymorphisms, OR: odds ratio (OR = 1 Exposure does not affect odds of outcome, OR > 1 Exposure caused with higher odds of outcome, OR < 1 Exposure caused with lower odds of outcome), CI: confidence interval, IVW: inverse-variance weighted. P _Effect_: Mendelian randomisation results in p-values; P _Heterogeneity_: Heterogeneity test; P _Intercept_: pleiotropy analysis; F _Statistic_: F values

### Body fat percentage

235 SNPs and 151 SNPs were separately collected to serve as IVs from the dataset of MRC-IEU consortium and the Neale Lab consortium’s dataset. The IVW results displayed that body fat percentage was caused with an elevated incidence of KOA ((OR = 1.57, 95% CI = 1.42–1.75) and (OR = 1.53, 95% CI = 1.37–1.72)) (Table 4).

**Table 4:** Mendelian randomization results in causal association between body fat percentage and KOA

Expose	Consortium	SNPs	Method	OR (95%CI)	*P*_Effect_	*P*_Heterogeneity_	*P*_Intercept_	F _Statistic_
Body Fat Percentage	MRC-IEU	235	IVW	1.57(1.42–1.75)	2.23 × 10^− 17^	0.97	0.46	53.86
			MR Egger	1.38(0.94-2.00)	9.86 × 10^− 2^	0.96		
			Weighted median	1.65(1.41–1.92)	2.11 × 10^− 10^			
			Simple mode	2.42(1.49–3.94)	4.61 × 10^− 4^			
			Weighted mode	1.84(1.22–2.76)	3.75 × 10^− 3^			
	Neale Lab	151	IVW	1.53(1.37–1.72)	3.64 × 10^− 13^	0.88	0.07	47.52
			MR Egger	0.99(0.61–1.62)	9.75 × 10^− 1^	0.91		
			Weighted median	1.57(1.32–1.85)	2.04 × 10^− 7^			
			Simple mode	1.48(0.89–2.45)	1.30 × 10^− 1^			
			Weighted mode	1.81(1.11–2.96)	1.91 × 10^− 2^			

SNPs: single nucleotide polymorphisms, OR: odds ratio (OR = 1 Exposure does not affect odds of outcome, OR > 1 Exposure caused with higher odds of outcome, OR < 1 Exposure caused with lower odds of outcome), CI: confidence interval, IVW: inverse-variance weighted. P _Effect_: Mendelian randomisation results in p-values; P _Heterogeneity_: Heterogeneity test; P _Intercept_: pleiotropy analysis; F _Statistic_: F values

### Waist circumference

A total of 215 SNPs was selected as IVs from the MRC-IEU consortium’s dataset. The causal assessment from the IVW results displayed that waist circumference had a causal relation with increased risks of KOA (OR = 1.60, 95% CI = 1.46–1.77). The Neale Lab consortium’s dataset was screened to select a total of 122 SNPs as IVs, while the GIANT consortium’s dataset selected 122 SNPs. The causal assessment from the IVW method revealed an OR (95% CI) value of 1.53 (1.37–1.71) and an OR (95% CI) value of 1.33 (1.14–1.55), respectively, exhibiting similar trends (Table 5).

**Table 5:** Mendelian randomization results in causal association between waist circumference and KOA

Expose	Consortium	SNPs	Method	OR (95%CI)	*P*_Effect_	*P*_Heterogeneity_	*P*_Intercept_	F _Statistic_
Waist circumference	MRC-IEU	215	IVW	1.60(1.46–1.77)	1.43 × 10^− 21^	1.00	0.65	50.96
			MR Egger	1.49(1.07–2.08)	1.87 × 10^− 2^	1.00		
			Weighted median	1.67(1.43–1.93)	1.87 × 10^− 11^			
			Simple mode	1.51(0.98–2.32)	6.09 × 10^− 2^			
			Weighted mode	1.70(1.22–2.38)	2.09 × 10^− 3^			
	Neale lab	122	IVW	1.53(1.37–1.71)	3.48 × 10^− 14^	0.99	0.79	48.16
			MR Egger	1.45(0.96–2.19)	8.01 × 10^− 2^	0.99		
			Weighted median	1.59(1.37–1.85)	1.64 × 10^− 9^			
			Simple mode	1.75(1.19–2.59)	5.61 × 10^− 3^			
			Weighted mode	1.73(1.18–2.53)	2.80 × 10^− 3^			
	GIANT	23	IVW	1.33(1.14–1.55)	3.00 × 10^− 4^	0.94	0.35	52.29
			MR Egger	1.73(0.98–3.08)	7.38 × 10^− 2^	0.95		
			Weighted median	1.48(1.21–1.80)	1.00 × 10^− 4^			
			Simple mode	1.52(1.05–2.20)	3.87 × 10^− 2^			
			Weighted mode	1.53(1.12–2.09)	1.40 × 10^− 2^			

SNPs: single nucleotide polymorphisms, OR: odds ratio (OR = 1 Exposure does not affect odds of outcome, OR > 1 Exposure caused with higher odds of outcome, OR < 1 Exposure caused with lower odds of outcome), CI: confidence interval, IVW: inverse-variance weighted. P _Effect_: Mendelian randomisation results in p-values; P _Heterogeneity_: Heterogeneity test; P _Intercept_: pleiotropy analysis; F _Statistic_: F values

### Hip circumference

259 SNPs were identified as IVs from the dataset of MRC-IEU consortium. IVW results showed hip circumference was causally related to an increased risk of KOA (OR = 1.39, 95% CI = 1.30–1.50). In the Neale Lab consortium’s dataset, a total of 166 SNPs were included as IVs after screening. The IVW results showed a similar trend (OR = 1.35, 95% CI = 1.24–1.46). A total of 30 SNPs were included as IVs in the GIANT consortium’s dataset and the IVW results showed an OR (95% CI) value of 1.34 (1.17–1.54), which were consistent with the former (Table 6).

**Table 6:** Mendelian randomization results in causal association between hip circumference and KOA

Expose	Consortium	SNPs	Method	OR (95%CI)	*P*_Effect_	*P*_Heterogeneity_	*P*_Intercept_	F _Statistic_
Hip circumference	MRC-IEU	259	IVW	1.39(1.29–1.50)	1.34 × 10^− 17^	0.88	0.83	56.18
			MR Egger	1.36(1.07–1.71)	1.15 × 10^− 2^	0.87		
			Weighted median	1.52(1.36–1.70)	3.55 × 10^− 13^			
			Simple mode	1.90(1.34–2.70)	3.50 × 10^− 4^			
			Weighted mode	1.65(1.26–2.16)	2.85 × 10^− 4^			
	Neale lab	166	IVW	1.35(1.24–1.46)	1.40 × 10^− 12^	0.97	0.97	50.33
			MR Egger	1.34(1.02–1.77)	3.80 × 10^− 2^	0.96		
			Weighted median	1.47(1.30–1.65)	1.88 × 10^− 10^			
			Simple mode	1.63(1.17–2.26)	4.55 × 10^− 3^			
			Weighted mode	1.59(1.18–2.14)	2.84 × 10^− 30^			
	GIANT	30	IVW	1.34(1.17–1.54)	2.02 × 10^− 5^	0.70	0.71	47.62
			MR Egger	1.22(0.74–2.02)	4.40 × 10^− 1^	0.66		
			Weighted median	1.48(1.23–1.79)	4.87 × 10^− 5^			
			Simple mode	1.52(1.06–2.19)	3.24 × 10^− 2^			
			Weighted mode	1.53(1.12–2.09)	1.26 × 10^− 2^			

SNPs: single nucleotide polymorphisms, OR: odds ratio (OR = 1 Exposure does not affect odds of outcome, OR > 1 Exposure caused with higher odds of outcome, OR < 1 Exposure caused with lower odds of outcome), CI: confidence interval, IVW: inverse-variance weighted. P _Effect_: Mendelian randomisation results in p-values; P _Heterogeneity_: Heterogeneity test; P _Intercept_: pleiotropy analysis; F _Statistic_: F values

### Waist–hip ratio

22 SNPs and 10 SNPs were selected as IVs in the GIANT consortium’s dataset and the within family GWAS consortium’s dataset, respectively. The IVW results disclosed WHR was caused with an decreased incidence of KOA ((OR = 0.86, 95% CI = 0.71–1.05) and (OR = 0.35, 95% CI = 0.01–8.31)) (Table 7).

**Table 7:** Mendelian randomization results in causal association between WHR and KOA

Expose	Consortium	SNPs	Method	OR (95%CI)	*P*_Effect_	*P*_Heterogeneity_	*P*_Intercept_	F _Statistic_
WHR	GIANT	22	IVW	0.86(0.71–1.05)	1.32 × 10^− 1^	0.37	0.75	39.55
			MR Egger	0.71(0.20–2.47)	5.91 × 10^− 1^	0.32		
			Weighted median	0.85(0.65–1.12)	2.54 × 10^− 1^			
			Simple mode	0.90(0.50–1.60)	7.16 × 10^− 1^			
			Weighted mode	0.88(0.51–1.53)	6.56 × 10^− 1^			
	Within family GWAS	10	IVW	0.35(0.01–8.31)	5.15 × 10^− 1^	0.16	0.61	62.69
			MR Egger	0.65(0.00-76.94)	4.70 × 10^− 1^	0.13		
			Weighted median	0.09(0.00-3.41)	1.99 × 10^− 1^			
			Simple mode	0.54(0.00-457.15)	8.63 × 10^− 1^			
			Weighted mode	0.10(0.00-7.98)	3.33 × 10^− 1^			

SNPs: single nucleotide polymorphisms, OR: odds ratio (OR = 1 Exposure does not affect odds of outcome, OR > 1 Exposure caused with higher odds of outcome, OR < 1 Exposure caused with lower odds of outcome), CI: confidence interval, IVW: inverse-variance weighted. P _Effect_: Mendelian randomisation results in p-values; P _Heterogeneity_: Heterogeneity test; P _Intercept_: pleiotropy analysis; F _Statistic_: F values

### Basal metabolic rate

329 SNPs and 225 SNPs were separately included as IVs in the MRC-IEU consortium’s dataset and the Neale Lab consortium’s dataset. The IVW results revealed BMR was causally related to an increased risk of KOA, ((OR = 1.39, 95% CI = 1.26–1.52) and (OR = 1.33, 95% CI = 1.21–1.46)) (Table 8).

**Table 8:** Mendelian randomization results in causal association between BMR and KOA

Expose	Consortium	SNPs	Method	OR (95%CI)	*P*_Effect_	*P*_Heterogeneity_	*P*_Intercept_	F _Statistic_
BMR	MRC-IEU	329	IVW	1.39(1.27–1.52)	2.97 × 10^− 12^	0.84	0.24	68.99
			MR Egger	1.22(0.97–1.54)	8.83 × 10^− 2^	0.85		
			Weighted median	1.40(1.21–1.63)	5.37 × 10^− 6^			
			Simple mode	1.65(1.02–2.68)	4.40 × 10^− 2^			
			Weighted mode	1.57(1.09–2.26)	1.49 × 10^− 2^			
	Neale lab	225	IVW	1.33(1.21–1.46)	3.75 × 10^− 9^	0.52	0.04	62.39
			MR Egger	1.05(0.82–1.35)	6.77 × 10^− 1^	0.58		
			Weighted median	1.39(1.20–1.62)	1.87 × 10^− 5^			
			Simple mode	1.67(1.07–2.61)	2.55 × 10^− 2^			
			Weighted mode	1.54(1.04–2.28)	3.08 × 10^− 2^			

SNPs: single nucleotide polymorphisms, OR: odds ratio (OR = 1 Exposure does not affect odds of outcome, OR > 1 Exposure caused with higher odds of outcome, OR < 1 Exposure caused with lower odds of outcome), CI: confidence interval, IVW: inverse-variance weighted. P _Effect_: Mendelian randomisation results in p-values; P _Heterogeneity_: Heterogeneity test; P _Intercept_: pleiotropy analysis; F _Statistic_: F values

### Meta-analysis results

**Fig. 2:**
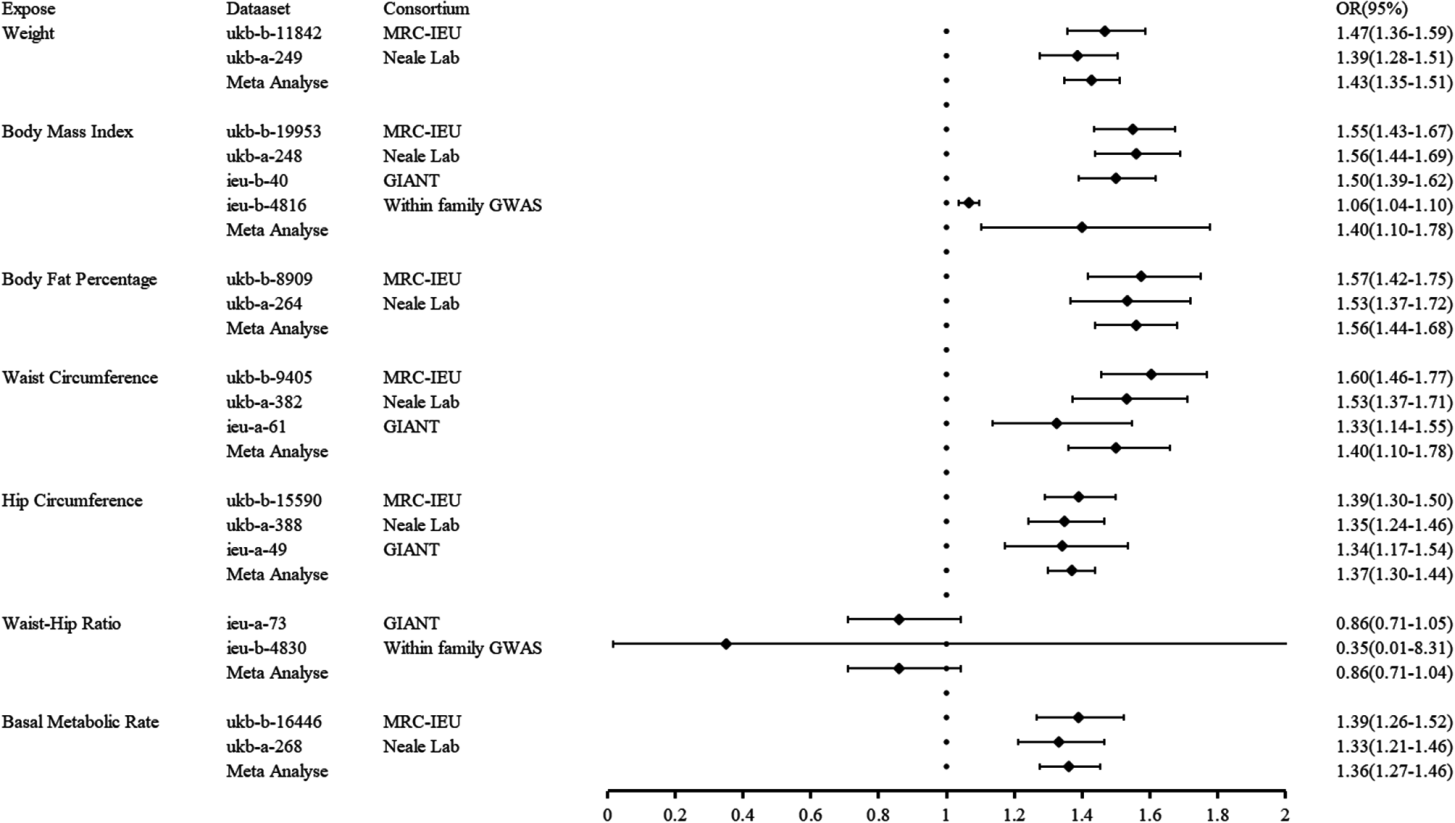
Meta-analysis of IVW method results from different data sources for body composition measurements

Meta-analysis was used to combine the results of the different datasets in Fig. 2. As expect, the results of OR trends were consistent with the ones from MR analyses For weight, meta-analysis showed *I*^2^ = 0.00% and a fixed effects model was used with an OR (95% CI) of 1.43 (1.35–1.51). For BMI, meta-analysis revealed *I*^2^ = 98.40%, so a random effects model was used with an OR of 1.40 (1.10–1.78). At the same time, excluding data sets from sources with large heterogeneity (within family GWAS consortium’s ieu-b-4816 dataset), meta-analysis examined *I*^2^ = 0.00%, hence a fixed effects model was used with an OR of 1.54 (1.47–1.61). For body fat percentage, as meta-analysis showed *I*^2^ = 0.00%, the fixed effects model was used with an OR of 1.56(1.44–1.68). For waist circumference, meta-analysis indicated *I*^2^ = 52.80%, therefore a random effects model was applied with an OR of 1.40 (1.10–1.78). For hip circumference, meta-analysis showed *I*^2^ = 0.00% and a fixed effects model was used with an OR of 1.37 (1.30–1.44). For WHR, *I*^2^ = 0.00% in meta-analysis and an OR of 0.86 (0.71–1.04) with a fixed effects model. For BMR, as meta-analysis showed *I*^2^ = 0.00%, the fixed effects model was used with an OR of 1.36 (1.27–1.46).

### Sensitivity analysis

The accuracy of the results between the body composition measurements and KOA were evaluated by sensitivity analysis. No significant heterogeneity was identified Tables 2, 3, 4, 5, 6, 7 and 8. Among the body composition measurements, the IVW intercept tests showed no evidence of pleiotropy, mostly (*P* > 0.05) Tables 2, 3, 4, 5, 6, 7 and 8, however, for weight and BMR analysis, the Neale Lab consortium’s dataset was found to have an insignificant pleiotropic analysis. The F value was greater than 40, proving no weak IV bias Tables 2, 3, 4, 5, 6, 7 and 8. Moreover, the MR estimation results predicted by leave-one-out analysis were not driven by specific SNPs.

## Discussion

Our study used MR to explore the causal association between body composition measurements from different data sources and KOA, body measurements mainly include weight, BMI, body fat percentage, waist circumference, hip circumference, WHR, and BMR. Meanwhile, in order to reduce the bias due to a single dataset, the content and quality of the article were increased to improve the credibility of the results. We used Meta-analysis to combine IVW results for the same body composition measurements from different data sources, and the combined results showed that the ORs for Weight, BMI, body fat percentage, waist circumference, hip circumference, WHR and BMR were 1.43 (1.35–1.51), 1.40 (1.10–1.78), 1.56 (1.44–1.68), 1.40 (1.10–1.78), 1.37 (1.30–1.44), 0.86 (0.71–1.04), and 1.36 (1.27–1.46), respectively. and it was found that body fat percentage may be a better response to KOA than the BMI.

There are a number of variables that influence the risk of KOA, with age, gender, and weight being the main factors [25, 26]. However, numerous early studies have demonstrated that there are multiple risk factors for the development and progression of KOA [27]. Zheng et al. found that obesity and overweight were significantly caused with the risk of KOA, with each 5 kg/m^2^ increase in BMI caused with a 35% increase in the risk of KOA [28]. In recent years, studies have revealed that other indicators regarding body composition measurements may also be risk factors of KOA while such indicators are also easy to measure and apply [29, 30]. Therefore, our study used MR to explore the causal analysis of weight, BMI, body fat percentage, waist circumference, hip circumference, WHR, and BMR with KOA. Wang et al. investigated the causal associations of obesity related anthropometric indicators and body compositions with knee and hip osteoarthritis [29, 30]. Contrary to the study, we used more datasets; in parallel, the sample was restricted to all sources of European origin which included males and females so as to avoid the influence of population issues on the results. The dataset was selected for the same exposure factor with the most recent year and a clear source, with a view to reducing the problem of bias through such a screening process. Finally, meta-analysis was used to combine the results of different dataset for the same indicator, in turn increasing the effect values to obtain reliable results.

Weight had been found to have a temporal causal effect in KOA in a large number of earlier studies [31, 32], but the results of the pleiotropic analysis of the Neale Lab consortium’s dataset in this study found a pleiotropic effect. The results of the MRC-IEU consortium’s dataset suggested a causal effect of weight in KOA, and the combined results had an OR of 1.43 (1.35–1.51), therefore weight in KOA might have a causal effect.

On this basis, we further explored the causality of other indicators for KOA. The causal relationship between BMI and KOA was demonstrated in a previous cohort study by Wills et al. and Funck-Brentano et al. [9, 23], in which the risk of KOA was found to accumulate into adulthood through exposure to high BMI. Our four datasets (five datasets existed in the search, one of which was the UK Biobank with exclusions because only one SNP was screened out) showed causal relationships between BMI and KOA, and the combined results showed an OR of 1.40 (1.10–1.78), but with high heterogeneity; exclusion of the heterogeneous datasets resulted in an OR of 1.54 (1.47–1.61), further clarifying the strength of the causal relationship.

Both datasets for body fat percentage suggested a causal relationship. Earlier studies by Long et al. found that both fat mass and fat percentage might be risk factors for KOA [33], and Karlsson et al. found a correlation between a higher percentage of fat mass and idiopathic KOA [34], further demonstrating a causal relationship between body fat percentage and KOA, with a combined OR of 1.56 (1.44–1.68), which was 0.16-times higher than the combined OR for BMI. When sensitivity analyses were performed using the leave-one-out method for BMI, the OR (95% CI) value after excluding the Within family GWAS dataset, which had the greatest heterogeneity, was 1.54 (1.30, 1.83), which was still smaller than the meta-analysis results for body fat percentage. This situation suggested that body fat percentage may be a better indicator for KOA than BMI, possibly because body fat percentage focused more on measuring body fat content. Whereas BMI was weight (kg)/height (m)^2^, which did not accurately describe the distribution of body fat and not distinguish between fat and muscle content. While muscle mass increase was a protective factor for KOA, fat mass increase was a KOA risk factor. Given that body fat percentage and BMI had a causal relationship with KOA, and both were able to account for the development and progression of KOA, using body fat percentage might be preferable to BMI.

Waist circumference and hip circumference was strongly correlated with KOA in the Vasilic-Brasnjevic S et al. study, and Holliday et al. found that both waist circumference and hip circumference were caused with the risk of developing KOA [30, 35]. Our study further demonstrated a causal relationship between waist circumference, hip circumference, and KOA in different datasets. However, the results of WHR in both datasets suggested that there was no causal relationship with KOA. In the previous studies, the findings on WHR were inconsistent. Holliday et al. found that WHR was not caused with KOA [35], but Lohmander et al. found an RR (risk ratio) of 2.2 for WHR [36]. On the other hand, Gandhi et al. found the RR of being obese [10], as determined by WHR if classified as obese by the BMI criteria, was 1.04 for men and 1.23 for women, suggesting that the causal relationship between WHR and KOA might be influenced by gender factors and requires further study.

In the two datasets for BMR analysis, the Neale Lab consortium’s dataset was found to have a statistically significant pleiotropic analysis, and the MRC-IEU consortium’s dataset suggested a causal relationship between BMR and KOA, with a combined OR of 1.36 (1.27–1.46). Therefore, BMR might have a causal effect in KOA. However, BMR was influenced by a number of factors, such as body surface area, growth stage, gender, nutrition, and functional status, thus was expected further analysis.

There were some limitations should be mentioned in this study. The sources of datasets for different physical measures were inconsistent, with four datasets present for some indicators and two datasets for others, which might have impacted the results. The discrepancy in findings due to gender differences evident in previous studies of the WHR, and failing to analyse gender separately, might also be insufficient in other measures. The datasets were all from European populations, so the findings may be applicable only in European populations and be of limited use for other populations. MR assumed a linear relationship between exposure factors and outcome factors, and did not apply if there was no linear relationship between the two.

## Conclusion

In summary, our study used MR to explore the causal relationships between weight, BMI, body fat percentage, waist circumference, hip circumference, WHR, and BMR in KOA. Additionally, we used meta-analysis to combine the results of different datasets and to enhance the strength of their causal associations. We found that weight and BMR might have a causal effect on KOA, but WHR did not. BMI, body fat percentage, waist circumference, and hip circumference had a causal relationship with KOA. Additionally, body fat percentage might be a better indicator of KOA than BMI.

## Data Availability

The data used in this study were publicly available. Weight: (dataset: ukb-b-11842, MRC-IEU, https://gwas.mrcieu.ac.uk/datasets/ukb-b-11842/; dataset: ukb-a-249, Neale Lab, https://gwas.mrcieu.ac.uk/datasets/ukb-a-249/); Body Mass Index: (dataset: ieu-b-40, GIANT, https://gwas.mrcieu.ac.uk/datasets/ieu-b-40/; dataset: ukb-b-19953, MRC-IEU, https://gwas.mrcieu.ac.uk/datasets/ukb-b-19953/; dataset: ukb-a-248, Neale Lab, https://gwas.mrcieu.ac.uk/datasets/ukb-a-248/; dataset: ieu-b-4816, Within family GWAS, https://gwas.mrcieu.ac.uk/datasets/ieu-b-4816/); Body Fat Percentage: (dataset: ukb-b-8909, MRC-IEU, https://gwas.mrcieu.ac.uk/datasets/ukb-b-8909/; dataset: ukb-a-264, Neale Lab, https://gwas.mrcieu.ac.uk/datasets/ukb-a-264/); Waist Circumference: (dataset: ukb-b-9405, MRC-IEU, https://gwas.mrcieu.ac.uk/datasets/ukb-b-9405/; dataset: ukb-a-382, Neale Lab, https://gwas.mrcieu.ac.uk/datasets/ukb-a-382/; dataset: ieu-a-61, GIANT, https://gwas.mrcieu.ac.uk/datasets/ieu-a-61/); Hip Circumference: (dataset: ukb-b-15590, MRC-IEU, https://gwas.mrcieu.ac.uk/datasets/ukb-b-15590/; dataset: ukb-a-388, Neale Lab, https://gwas.mrcieu.ac.uk/datasets/ukb-a-388/; dataset: ieu-a-49, GIANT, https://gwas.mrcieu.ac.uk/datasets/ieu-a-49/); Waist-Hip Ratio: (dataset: ieu-a-73, GIANT, https://gwas.mrcieu.ac.uk/datasets/ieu-a-73/; dataset: ieu-b-4830, Within family GWAS, https://gwas.mrcieu.ac.uk/datasets/ieu-b-4830/); Basal Metabolic Rate: (dataset: ukb-b-16446, MRC-IEU, https://gwas.mrcieu.ac.uk/datasets/ukb-b-16446/; dataset: ukb-a-268, Neale Lab, https://gwas.mrcieu.ac.uk/datasets/ukb-a-268/); Knee Osteoarthritis: (dataset: ebi-a-GCST007090 UK, Biobank, https://gwas.mrcieu.ac.uk/datasets/ebi-a-GCST007090/)

## Abbreviations

KOA: Osteoarthritis Knee
BMI: Body Mass Index
WHR: Waist Hip Ratio
BMR: Basal Metabolic Rate
IVW: Inverse Variance Weighted
MR: Mendelian Randomization
IV: Instrumental Variable
SNPs: Single Nucleotide Polymorphisms
OR: Odds Ratio
CI: Confidence Interval

## References

[1] Brophy RH, Fillingham YA, AAOS Clinical Practice Guideline Summary (2022). Management of Osteoarthritis of the knee (nonarthroplasty), Third Edition. J Am Acad Orthop Surg.

[2] Giorgino R, Albano D, Fusco S, Peretti GM, Mangiavini L, Messina C. Knee osteoarthritis: Epidemiology, Pathogenesis, and mesenchymal stem cells: what else is New? An update. Int J Mol Sci 2023; 24(7). 10.3390/ijms24076405 PMC10094836 37047377

[3] Georgiev T, Angelov AK (2019). Modifiable risk factors in knee osteoarthritis: treatment implications. Rheumatol Int.

[4] Ma XL, Hu YC, Wang KZ (2022). Chinese clinical practice guidelines in treating knee osteoarthritis by Periarticular Knee Osteotomy. Orthop Surg.

[5] Safiri S, Kolahi AA, Smith E, Hill C, Bettampadi D, Mansournia MA, Hoy D, Ashrafi-Asgarabad A, Sepidarkish M, Almasi-Hashiani A (2020). Global, regional and national burden of osteoarthritis 1990–2017: a systematic analysis of the global burden of Disease Study 2017. Ann Rheum Dis.

[6] Turkiewicz A, Petersson IF, Björk J, Hawker G, Dahlberg LE, Lohmander LS, Englund M (2014). Current and future impact of osteoarthritis on health care: a population-based study with projections to year 2032. Osteoarthritis Cartilage.

[7] Kulkarni K, Karssiens T, Kumar V, Pandit H (2016). Obesity and osteoarthritis. Maturitas.

[8] Reyes C, Leyland KM, Peat G, Cooper C, Arden NK, Prieto-Alhambra D (2016). Association between Overweight and Obesity and risk of clinically diagnosed knee, hip, and Hand Osteoarthritis: a Population-based Cohort Study. Arthritis Rheumatol.

[9] Wills AK, Black S, Cooper R, Coppack RJ, Hardy R, Martin KR, Cooper C, Kuh D (2012). Life course body mass index and risk of knee osteoarthritis at the age of 53 years: evidence from the 1946 British birth cohort study. Ann Rheum Dis.

[10] Gandhi R, Dhotar H, Tsvetkov D, Mahomed NN (2010). The relation between body mass index and waist-hip ratio in knee osteoarthritis. Can J Surg.

[11] Gill SV, Hicks GE, Zhang Y, Niu J, Apovian CM, White DK (2017). The association of waist circumference with walking difficulty among adults with or at risk of knee osteoarthritis: the Osteoarthritis Initiative. Osteoarthritis Cartilage.

[12] Zhang W, Doherty M, Peat G, Bierma-Zeinstra MA, Arden NK, Bresnihan B, Herrero-Beaumont G, Kirschner S, Leeb BF, Lohmander LS (2010). EULAR evidence-based recommendations for the diagnosis of knee osteoarthritis. Ann Rheum Dis.

[13] Sekula P, Del GMF, Pattaro C, Köttgen A (2016). Mendelian randomization as an Approach to assess causality using Observational Data. J Am Soc Nephrol.

[14] Skrivankova VW, Richmond RC, Woolf B, Yarmolinsky J, Davies NM, Swanson SA, Vanderweele TJ, Higgins J, Timpson NJ, Dimou N (2021). Strengthening the reporting of Observational studies in Epidemiology using mendelian randomization: the STROBE-MR Statement. JAMA.

[15] Katan MB (1986). Apolipoprotein E isoforms, serum cholesterol, and cancer. Lancet.

[16] van Oort S, Beulens J, van Ballegooijen AJ, Grobbee DE, Larsson SC (2020). Association of Cardiovascular Risk factors and lifestyle behaviors with hypertension: a mendelian randomization study. Hypertension.

[17] Yuan S, Larsson SC (2021). Assessing causal associations of obesity and diabetes with kidney stones using mendelian randomization analysis. Mol Genet Metab.

[18] Zhang Z, Wang M, Yuan S, Liu X. Alcohol, Coffee, and milk intake in relation to Epilepsy Risk. Nutrients. 2022; 14(6). 10.3390/nu14061153 PMC8951548 35334809

[19] Davies NM, Holmes MV, Davey SG (2018). Reading mendelian randomisation studies: a guide, glossary, and checklist for clinicians. BMJ.

[20] Gupta V, Walia GK, Sachdeva MP (2017). Mendelian randomization’: an approach for exploring causal relations in epidemiology. Public Health.

[21] Boef AG, Dekkers OM, le Cessie S (2015). Mendelian randomization studies: a review of the approaches used and the quality of reporting. Int J Epidemiol.

[22] Tachmazidou I, Hatzikotoulas K, Southam L, Esparza-Gordillo J, Haberland V, Zheng J, Johnson T, Koprulu M, Zengini E, Steinberg J (2019). Identification of new therapeutic targets for osteoarthritis through genome-wide analyses of UK Biobank data. Nat Genet.

[23] Funck-Brentano T, Nethander M, Movérare-Skrtic S, Richette P, Ohlsson C (2019). Causal factors for knee, hip, and Hand Osteoarthritis: a mendelian randomization study in the UK Biobank. Arthritis Rheumatol.

[24] Xie J, Huang H, Liu Z, Li Y, Yu C, Xu L, Xu C (2023). The associations between modifiable risk factors and nonalcoholic fatty liver disease: a comprehensive mendelian randomization study. Hepatology.

[25] Neogi T, Zhang Y (2013). Epidemiology of osteoarthritis. Rheum Dis Clin North Am.

[26] Pradelli L, Sinigaglia T, Migliore A, Checchia GA, Franceschi F, Frediani B, Iannone F, Romanini E (2021). Non-surgical treatment of knee osteoarthritis: Multidisciplinary Italian Consensus on best practice. Ther Clin Risk Manag.

[27] Sun X, Zhen X, Hu X, Li Y, Gu S, Gu Y, Dong H. Osteoarthritis in the Middle-aged and Elderly in China: prevalence and influencing factors. Int J Environ Res Public Health. 2019; 16(23). 10.3390/ijerph16234701 PMC6926632 31779104

[28] Zheng H, Chen C (2015). Body mass index and risk of knee osteoarthritis: systematic review and meta-analysis of prospective studies. Bmj Open.

[29] Holmberg S, Thelin A, Thelin N (2005). Knee osteoarthritis and body mass index: a population-based case-control study. Scand J Rheumatol.

[30] Vasilic-Brasnjevic S, Marinkovic J, Vlajinac H, Vasiljevic N, Jakovljevic B, Nikic M, Maksimovic M (2016). Association of body mass index and waist circumference with severity of knee osteoarthritis. Acta Reumatol Port.

[31] Katz JN, Arant KR, Loeser RF (2021). Diagnosis and treatment of hip and knee osteoarthritis: a review. JAMA.

[32] Panunzi S, Maltese S, De Gaetano A, Capristo E, Bornstein SR, Mingrone G (2021). Comparative efficacy of different weight loss treatments on knee osteoarthritis: a network meta-analysis. Obes Rev.

[33] Long H, Xie D, Zeng C, Wei J, Wang Y, Yang T, Xu B, Qian Y, Li J, Wu Z (2019). Association between body composition and osteoarthritis: a systematic review and meta-analysis. Int J Rheum Dis.

[34] Karlsson MK, Magnusson H, Cöster M, Karlsson C, Rosengren BE (2015). Patients with knee osteoarthritis have a phenotype with higher bone mass, higher fat mass, and lower lean body mass. Clin Orthop Relat Res.

[35] Holliday KL, Mcwilliams DF, Maciewicz RA, Muir KR, Zhang W, Doherty M (2011). Lifetime body mass index, other anthropometric measures of obesity and risk of knee or hip osteoarthritis in the GOAL case-control study. Osteoarthritis Cartilage.

[36] Lohmander LS, Gerhardsson DVM, Rollof J, Nilsson PM, Engström G (2009). Incidence of severe knee and hip osteoarthritis in relation to different measures of body mass: a population-based prospective cohort study. Ann Rheum Dis.

